# Hydrolyzable tannins in local Thai plants: Potential applications as poultry feed supplements: A systematic review

**DOI:** 10.14202/vetworld.2025.2051-2063

**Published:** 2025-07-27

**Authors:** Tanakamol Mahawan, Pornchai Pornpanom, Surya Nur Rahmatullah, Tuempong Wongtawan, Thotsapol Thomrongsuwannakij

**Affiliations:** 1 Akkhraratchakumari Veterinary College, Walailak University, Nakhon Si Thammarat 80160, Thailand; 2 One Health Research Center, Walailak University, Nakhon Si Thammarat 80160, Thailand; 3 Research Center for Theoretical Simulation and Applied Research in Bioscience and Sensing, Walailak University, Nakhon Si Thammarat 80160, Thailand; 4 Department of Animal Husbandry, Faculty of Agriculture, Mulawarman University, Samarinda 75119, Indonesia

**Keywords:** antibiotic alternatives, antimicrobial activity, ellagic acid, gallic acid, gut health, hydrolyzable tannins, *Manihot esculenta*, poultry feed, *Senna siamea*, sustainable agriculture, Thai plants

## Abstract

**Background and Aim:**

The global shift toward antibiotic-free poultry production necessitates sustainable alternatives to conventional growth promoters. Hydrolyzable tannins (HTs) from plants have shown antimicrobial, antioxidant, and gut-modulatory effects, making them promising feed additives. However, reliance on imported tannins from temperate species limits access for tropical producers, especially in Thailand. This study aimed to systematically evaluate locally available Thai plant species as alternative sources of HTs for poultry feed, with a focus on their biological activities, economic feasibility, and practical integration potential.

**Materials and Methods:**

A systematic literature search (2020–2024) was conducted using PubMed, ScienceDirect, and the Thai citation index. Studies assessing Thai plant-derived HTs and their antimicrobial, antioxidant, and gut health effects were included in the study. A total of 21 studies covering 24 plant species were analyzed. Data extraction included tannin type, target microbes, experimental outcomes, and yield/economic feasibility, assessed through a validated scoring system.

**Results:**

Gallic and ellagic acids were the predominant bioactive compounds reported. HT-rich extracts demonstrated strong antimicrobial effects against 19 pathogenic bacterial species and enhanced the growth of beneficial gut microbiota, including *Lactobacillus* spp. and *Faecalibacterium prausnitzii*. *Manihot esculenta* (cassava) and *Senna siamea* (Siamese cassia) emerged as top candidates based on both bioactivity and economic feasibility. *In vivo* studies, although limited, supported their positive impact on gut health in broilers.

**Conclusion:**

Thai HT-rich plants, particularly cassava and Siamese cassia, show strong potential as sustainable feed additives in poultry production. These species offer dual benefits: antimicrobial and gut-modulatory effects and reduced reliance on expensive imported tannins. However, more standardized extraction protocols and large-scale *in vivo* trials are essential to validate efficacy, optimize dosage, and ensure feed safety.

## INTRODUCTION

Tannins are a structurally diverse group of naturally occurring phenolic compounds, primarily recognized for their ability to bind and precipitate proteins. They are broadly classified into two types: Hydrolyzable tannins (HTs) and condensed tannins (CTs) [1]. Each type plays a distinct role in animal nutrition, with specific relevance to monogastric and ruminant species. HTs are particularly beneficial for monogastric animals, such as poultry and swine, due to their capacity to support gut health and improve nutrient absorption [2]. Within the gastrointestinal tract, HTs are hydrolyzed to release active metabolites such as gallic acid and ellagic acid—compounds with strong antimicrobial and antioxidant properties [3]. These effects contribute to a balanced gut microbiome and enhanced nutrient uptake. In contrast, CTs are more suited to ruminants like cattle, as they reduce ruminal protein degradation by forming stable protein complexes, thereby improving protein utilization and productivity [4].

Globally, chicken is the most consumed meat, driving continued expansion in poultry production [5]. Growing consumer awareness of food safety and sustainability has intensified the demand for antibiotic-free poultry products. This trend is further accelerated by the rising threat of antimicrobial resistance (AMR), which affects both broilers and layers, posing a significant global health concern [6]. In Southeast Asia, where antibiotic use is often inadequately regulated, the poultry industry is facing increasing pressure to eliminate antibiotics from its production systems [7]. Despite bans on antibiotic growth promoters (AGPs), there is a scarcity of effective, locally adapted alternatives in tropical regions. Phytogenic additives such as tannins have shown promise but remain underexplored, particularly in the context of locally available plant sources. Thailand, currently the third-largest global exporter of chicken, lacks comprehensive data on antibiotic usage in poultry farming [8].

The global tannin market is dominated by temperate species, such as chestnut and quebracho, with international trade valued in the tens of millions of dollars [9]. This dependence on imports renders Thailand’s poultry sector vulnerable to price volatility and supply chain disruptions, underscoring the need to identify domestic sources of tannin. Due to their wide-ranging bioactivities, HTs are increasingly recognized as viable alternatives to synthetic feed additives. Their bioactive constituents can inhibit both Gram-positive and Gram-negative bacteria, thereby promoting gut health and enhancing disease resistance in poultry [10]. As regulatory restrictions on AGPs tighten and concerns about AMR escalate [11], tannins offer a sustainable alternative that aligns with both industry demands and consumer expectations [12].

By 2023, Thailand had become the world’s third-largest chicken exporter, following Brazil and the United States, and had begun adopting antibiotic-free production models to meet international standards [13]. However, the lack of effective, economically viable alternatives to AGP remains a critical challenge. The poultry sector’s continued reliance on imported tannin extracts–primarily derived from chestnut and quebracho–drives up production costs and limits access for smallholder farmers. Leveraging Thailand’s abundant plant biodiversity to identify indigenous HT sources could offer a cost-effective and sustainable alternative, thereby reducing import dependence and strengthening local agricultural systems. As a tropical country, Thailand hosts a diverse array of plant species with potential utility in tannin production [14]. Although the beneficial effects of tannins on gut health and microbial balance are well-documented, their targeted application in poultry diets – especially in terms of HT composition, phytochemical characterization, and *in vivo* efficacy – remains insufficiently studied. Furthermore, a systematic assessment of Thai plant species as feasible HT sources for poultry feed is currently lacking. Previous research by Singh and Kumar [15] has established the functional benefits of tannins, including their ability to modulate intestinal microbial ecosystems and improve productivity. However, a comprehensive evaluation of local botanical sources is urgently needed.

While HTs have garnered increasing attention for their antimicrobial, antioxidant, and gut health-enhancing properties in poultry nutrition, the current body of research is predominantly centered around tannin sources from temperate regions – most notably chestnut (*Castanea sativa*) and quebracho (*Schinopsis* spp.). These imported extracts dominate the global market, creating dependency that may be economically and logistically unsustainable, especially for tropical countries like Thailand. Despite Thailand’s rich biodiversity and traditional ethnoveterinary knowledge, a comprehensive evaluation of indigenous plant species as viable sources of HTs remains largely unexplored. Most available studies have focused on total phenolic content rather than specifically quantifying HTs or assessing their *in vivo* effects on poultry. Moreover, there is considerable variability in extraction protocols, plant part selection, and reported outcomes, which limits reproducibility and practical application. The lack of standardized methodologies and insufficient data on the economic feasibility of integrating these local plants into commercial poultry feed further hinders their adoption. Therefore, a critical research gap exists in systematically identifying, characterizing, and evaluating local Thai HT-rich plants for their efficacy and scalability in antibiotic-free poultry production systems.

This systematic review aims to comprehensively assess the potential of locally available Thai plant species as alternative sources of HTs for use in poultry feed. Specifically, the objectives are threefold: (1) to identify and catalogue Thai plants with documented HT content and bioactive compounds such as gallic and ellagic acids; (2) to evaluate the antimicrobial, antioxidant, and gut-modulating effects of these plant-derived extracts through both *in vitro* and *in vivo* findings; and (3) to analyze the economic feasibility and scalability of these plants for integration into poultry feed formulations. By synthesizing existing scientific evidence with ethnobotanical knowledge and economic data, this review seeks to provide a foundational reference for researchers, policymakers, and feed manufacturers interested in developing cost-effective, locally sourced, and sustainable feed additives. The findings are intended to support Thailand’s transition to antibiotic-free poultry production while offering a replicable framework for similar initiatives in other tropical regions.

## MATERIALS AND METHODS

### Ethical approval

This systematic review was conducted in accordance with the Preferred Reporting Items for Systematic Reviews and Meta-Analyses (PRISMA) 2020 guidelines [16], incorporating all 27 checklist items as outlined in supplementary data 1.

### Study period and location

The literature search was conducted in November 2024 at Walailak University, Nakhon Si Thammarat, Thailand.

### Protocol registration

The study protocol was prospectively registered with the Open Science Framework (OSF) under the identifier https://doi.org/10.17605/OSF.IO/SJ3KD.

### Population, intervention, comparison, outcome, and study design [PICOS] framework)

The scope of this review was defined using the PICOS framework:

- Population: Poultry species and relevant animal models associated with poultry nutrition.
- Intervention: Administration of HTs derived from Thai local plants.
- Comparator: Synthetic tannin formulations, antibiotic treatments, or untreated control groups.
- Outcomes: Antimicrobial activity, modulation of gut health, and cost-effectiveness assessments.
- Study design: Experimental (*in vitro* and *in vivo*) and observational studies.

### Information sources and search strategy

A comprehensive literature search was performed in three databases: PubMed, ScienceDirect, and the Thai Citation Index (TCI). PubMed was selected for its robust biomedical indexing, whereas ScienceDirect provided a wide range of agricultural and veterinary science content. The TCI was included to capture region-specific studies not indexed internationally. The final search was conducted on November 15, 2024. Articles in both English and Thai were eligible for inclusion. The following Boolean search strategies were employed:

- PubMed: (“Thailand” OR “Thai plant” OR “Thai tree”) AND (“hydrolyzable tannin” OR “tannin” OR “gallic” OR “ellagic”) NOT (“review”)
- ScienceDirect: (“Thai plant” OR “Thai tree”) AND (“hydrolyzable tannin” OR “tannin” OR “gallic” OR “ellagic”) AND (“antimicrobial” OR “gut”)
- TCI: “tannin” (filtered for antimicrobial-related studies due to limitations in advanced search capabilities).

The complete search strategy, including filters and syntax configurations, is provided in supplementary data 3.

### Study selection process

A multiphase screening process was employed to ensure rigorous selection. Initial screening involved title-based filtering for studies that referenced Thai plants and were relevant to poultry nutrition. Articles that ambiguously described their objectives or included antimicrobial testing without clear titles were marked as “remarked papers” for further review.

Abstracts were subsequently assessed to confirm the presence of health-related endpoints, such as antimicrobial or gut health effects. A full-text review was then conducted to confirm the inclusion of HTs (e.g., gallic or ellagic acid), the use of specific microbial assays, and the relevance to animal or human health, rather than non-health industrial applications.

Two reviewers (TM and PP) independently performed full-text screening. Both were trained on inclusion/exclusion criteria, and the review process was blinded to authorship and institutional affiliations. Disagreements were resolved through discussion; if unresolved, a third reviewer (TT) served as the adjudicator. References were managed using EndNote 20 (Clarivate Analytics, Philadelphia, PA, USA) to ensure systematic documentation and retrieval.

### Inclusion criteria

1. Original research using experimental, analytical, or observational designs involving Thai plants.
2. Studies reporting the presence or application of HTs (e.g., tannic, gallic, or ellagic acid).
3. Studies assessing antimicrobial, antioxidant, or gut health effects in animals or humans.
4. Publications dated between 2020 and 2024.
5. Reports containing at least one quantifiable HT-related measurement.

### Exclusion criteria

1. Studies are limited to phytochemical or nutritional profiling without biological testing.
2. Use of HTs for non-health purposes (e.g., textiles, food preservation, and adhesives).
3. Abstracts, conference proceedings, theses, preprints, and reviews.
4. Studies for which the full text was not accessible.

### Selection of publications

The final article selection involved multiple evaluative steps to ensure comprehensiveness and reduce bias. Articles with misleading titles were flagged for detailed abstract and full-text analysis. Eligible studies were those explicitly investigating HTs’ biological effects (especially antimicrobial activity), identified microbial species, and health-related objectives. Each study had to demonstrate relevance to application, particularly in the context of poultry feed development. Inter-reviewer agreement was assessed using Cohen’s Kappa, yielding a value of 0.84 (95% confidence interval: 0.71–0.98, n = 76), indicating excellent consistency.

### Data extraction and management

Data from eligible studies were independently extracted by two reviewers (TM and PP) using a structured Excel spreadsheet Office 365 version 2203 (Microsoft Office, Washington, USA). Extracted variables included:

1. Plant common name
2. Scientific name
3. Thai/local name
4. Plant part used (e.g., leaves, bark, roots)
5. Type of tannin identified (e.g., HT, gallic acid, ellagic acid)
6. Documented biological effects (e.g., antibacterial, antioxidant)
7. Target microbial species
8. Traditional or prospective applications

Discrepancies were reconciled through discussion, with a third reviewer (TT) verifying data integrity. The final dataset was used for narrative synthesis. Double data entry was employed to ensure consistency and accuracy, as detailed in supplementary data 2.

### Risk of bias assessment

Methodological rigor and bias across studies were assessed using the ROBIS tool version 1.2 (University of Bristol, UK) [17]. Independent assessments by two reviewers were conducted to evaluate study eligibility, selection process, data collection, and synthesis. Discrepancies were resolved through consensus.

### Data synthesis and integration

Due to heterogeneity in study designs, interventions, and outcome measures, meta-analysis was not feasible. Instead, a narrative synthesis was performed, following the framework by Popay *et al*. [18]. The synthesis process included:

- Classification of plant species based on the part used
- Qualitative comparison of bioactive compounds (e.g., gallic acid, ellagic acid)
- Integration of reported antimicrobial, antioxidant, and gut health effects
- Aggregation of evidence regarding inhibition of pathogenic microbes and promotion of beneficial gut flora.

### Economic feasibility and yield assessment

To evaluate the practical applicability of Thai HT-rich plants in poultry feed, a scoring system was developed based on two criteria: Yield and economic feasibility, each rated on a 1–5 scale (Table 1). The final feasibility score was the average of the two. Data were sourced from local agricultural reports, Food and Agriculture Organization corporate statistical database [19], and other global databases when Thai data were unavailable. The system was reviewed by subject-matter experts to ensure its validity. This approach provided a realistic assessment of which plants could be sustainably scaled for commercial feed production.

**Table 1:** Yield and economic feasibility scoring criteria. Scores represent the suitability of plants for large-scale production based on yield and economic feasibility, with higher scores indicating greater potential for cost-effective utilization.

Score	Yield score	Economic feasibility score
5	Very high - The plant produces a very high yield, making it highly suitable for large-scale production.	Highly feasible: The plant is highly cost-effective, with low production costs and high market value.
4	High - This plant produces high yields suitable for significant production levels.	Feasible: The plant is cost-effective, with reasonable production costs and good market value.
3	Moderate: The plant produces a moderate yield, which is acceptable for medium-scale production.	Moderately feasible: The plant has moderate production costs and market value.
2	Low - This plant produces low yield, limiting its suitability for large-scale production.	Less feasible: The plant has higher production costs and lower market value.
1	Very low - This plant produces a very low yield, making it unsuitable for large-scale production.	Not feasible: The plant is not cost-effective, with high production costs and low market value.

## RESULTS

### Characteristics of included studies

**Figure 1:**
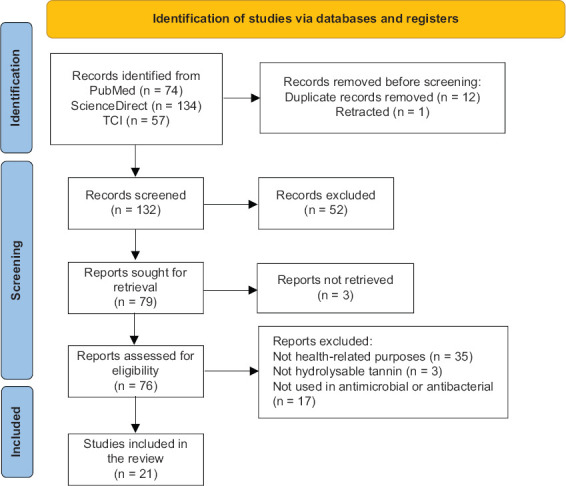
Preferred reporting items for systematic reviews and meta-analyses flowchart showing the identification, screening, and inclusion processes of the systematic reviews.

A total of 265 articles were initially retrieved through comprehensive searches of public databases. Following the removal of 13 records, comprising 12 duplicates and one retracted publication, 132 articles were retained for title and abstract screening. After applying preliminary inclusion criteria, 76 articles were shortlisted for full-text evaluation. Ultimately, 21 studies met all eligibility requirements and were included in the final synthesis, as illustrated in Figure 1. These studies investigated 24 unique plant species native to Thailand, detailed in Table 2 [20–40].

**Table 2:** Characteristics of included studies on tannin-containing Thai plants, including scientific name, common name, and plant parts used.

No.	Reference	Scientific name	Common name	Parts
1	Wacharatewinkul *et al*. [20]	*Tacca leontopetaloides (L.) Kuntze.*	Polynesian Arrowroot	Peel
2	Seekhaw *et al*. [21]	*Flacourtia indica*	Governor’s plum	Fruits
3	Pimrote *et al*. [22]	*Shorea roxburghii G. Don*	Shorea	Peel, Roots, and Leaves
4	Limsuwan *et al*. [23]	*Quercus infectoria*	Aleppo oak	Nutgall
5	Mahboob *et al*. [24]	*Leea indica (Burm. f.) Merr.*	Common tree-vine	Leaves
6	Pintatum *et al*. [25]	*Zingiber kerrii Craib*	Zingiber kerrii	Rhizomes, Flowers, and Leaves
7	Singhapol *et al*. [26]	*Citrus aurantifolia*	Citrus	Peel
8	Khoontawad *et al*. [27]	*Combretum quadrangulare Kurz*	Bushwillows	Branches, Barks, Fruits, and Leaves
9	Tantratian *et al*. [28]	*Syzygium cumini and Mentha cordifolia*	Java Plum and Mint	Seed and Leaves
10	Charoensiddhi *et al*. [29]	*Vigna radiata L.*	Mung bean	Seed coat
11	Leesombun *et al*. [30]	*Punica granatum L.*	Pomegranate	Bark
12	Chansiw *et al*. [31]	*Polygonum odoratum*	Vietnamese Coriander	Leaves
13	Sripontan *et al*. [32]	*Manihot esculenta*	Cassava	Leaves
14	Kwandee*et al*. [33]	*Phyllanthus emblica, Terminalia bellirica,and Terminalia chebula*	Triphala: Indian gooseberry, beleric myrobalan, and black myrobalan	Fruits
15	Sainakham *et al*. [34]	*Curcuma aromatica*	Wild turmeric	Rhizome
16	Sam-Ang *et al*. [35]	*Morinda citrifolia*	Noni	Roots
17	Thongdonphum *et al*. [36]	*Nymphaea pubescens*	Pink water lily	Leaves
18	Thanasut *et al*. [37]	*Eclipta prostrata (L.) L.*	Kameng or false daisy	Leaves
19	Ogunniran *et al*. [38]	*Senna siamea*	Siamese cassia	Leaves
20	Wachiradejkul *et al*. [39]	*Ocimum sanctum*	Holy basil	Flower
21	Cheong *et al*. [40]	*Parkia speciosa*	Bitter bean	Pods

### Risk of bias assessment

The risk of bias across the included studies was assessed as low in all evaluated domains. The eligibility criteria were clearly defined, the selection and data extraction processes were rigorously conducted, and the synthesis approach accounted for study heterogeneity and potential confounding factors. Interpretations were balanced and avoided overstating significance. Overall, the review process demonstrated strong methodological integrity, as further detailed in supplementary data 4.

### Plant species included in the review

The 24 Thai plant species examined across the 21 included studies were analyzed for their bioactive properties derived from specific plant parts such as leaves, bark, roots, fruits, rhizomes, peels, and flowers (Table 2). Most studies focused on a single plant part – primarily leaves – although some species, including *Shorea roxburghii* (Shorea) and *Combretum quadrangulare* (Bushwillows), were studied across multiple parts (e.g., bark, roots, and leaves). Others, such as *Ocimum sanctum* (Holy basil) and *Citrus aurantifolia* (Citrus), were evaluated mainly for their floral and peel-derived extracts, respectively.

### Plant-based sources of tannins and their biological activities

Variations in plant parts used contributed to differences in HT concentration and composition. HT-rich extracts demonstrated a broad range of biological activities, including antimicrobial, anti-inflammatory, antioxidant, and gut health-promoting effects. Notably, bioactive compounds such as gallic acid and ellagic acid were frequently identified as key contributors to these effects (Table 3) [20–40].

**Table 3:** Plant-based sources of tannins and their biological effects in poultry and other animals, including the type of tannin and experiment type.

Effect	Plant name	Tannin	Experiment type	Hydrolyzable tannin concentration	Unit
Antibacterial	Polynesian arrowroot [20]	Gallic acid	*In vitro*	2.62 (flour extracts) 8.68 (peel extracts)	mg TAE/g
	Aleppo oak [23]	Gallic acid and Ellagic acid	*In vitro*	31.25–62.5 (ethanol extract) 31.25–250 (water extract)	μg/mL
	Citrus [26]	Gallic acid	*In vivo* (Shrimps)	25–400 (peel extracts)	mg/mL
	Java plum and Mint [28]	Gallic acid	*In vitro*	188.5 (dried extract)	mg/g
	Pink water lily [36]	Gallic acid	*In vitro*	0.600–3.21	% w/w
	Bushwillows [37]	Ellagic acid	*In vitro*	Present	(qualitative)
Anti-inflammatory and antibacterial	Vietnamese Coriander [31]	Gallic acid	*In vitro*	52.59 ± 0.58	mg GAE/g
	Wild turmeric [34]	Gallic acid	*In vitro*	2.67 ± 0.17–19.36 ± 0.26	mg GAE/g
Antioxidant and antimicrobial	Siamese cassia [38]	Gallic acid	*In vitro*	13.25 ± 0.03	mg GAE/g
	Shorea [22]	Ellagic acid	*In vitro*	23.33 (bark), 19.35 (root), 18.19 (leaf)	µg/mL
	Governor’s plum [21]	Gallic acid and Ellagic acid	*In vitro*	0.3044 ± 0.0067	mg GAE/g
	Noni [35]	Gallic acid	*In vitro*	Strongly present	(qualitative)
	Zingiber kerrii [25]	Gallic acid	*In vitro*	Present	(qualitative)
	Pomegranate [30]	Gallic acid	*In vitro*	574.64 (bark), 242.60 (peel)	mg GAE/g
Antibacterial and improve broiler gut health	Cassava [32]	Gallic acid	*In vivo* (Broilers)	10, 20 and 30	mg/l
Antioxidant and intestinal barrier support	Holy basil [39]	Chlorogenic acid and Gallic acid	*In vitro*	4.86 ± 0.02	mg GAE/g
Microbiome modulation	Triphala [33]	Gallic acid	*In vitro*	177.27 ± 10.09	mg GAE/mL
Anti-inflammatory, anti-diabetic, and antibacterial	Bitter bean [40]	Gallic acid	*In vitro*	84.53 ± 9.40	mg GAE/g
Anti-inflammatory, anti-diabetic, and gut microbiota modulation	Mung bean [29]	Gallic acid	*In vitro*	320.50 ± 25.66	mg GAE/g
Coccidia sporulation inhibition	Kameng or false daisy [37]	Gallic acid and Ellagic acid	*In vitro*	70–70,000	mg/L
Anti-acanthamoebic	Common tree-vine [24]	Gallic acid	*In vitro*	25–100	μg/mL

TAE=Tannic Acid Equivalents; tannin content measured relative to tannic acid. GAE=Gallic acid equivalents; total phenolic content measured relative to gallic acid

Several plant species exhibited potent antibact-erial activity. For instance, *Tacca leontopetaloides* (Polynesian arrowroot), *Quercus infectoria* (Aleppo oak), and *Flacourtia indica* (Governor’s plum) were effective against *Escherichia coli*, *Staphylococcus aureus*, and *Pseudomonas aeruginosa*. Other species, such as *Polygonum odoratum* (Vietnamese coriander) and *Curcuma aromatica* (Wild turmeric), displayed both antibacterial and anti-inflammatory properties. Antiparasitic effects were also reported in some species. In addition, antioxidant activity was noted in *Senna siamea* (*Siamese cassia*), *S. roxburghii* (Shorea), and *Punica granatum* (Pomegranate), suggesting potential benefits for poultry immune function and oxidative stress mitigation.

Two *in vivo* studies specifically assessed the impact of *Manihot esculenta* (Cassava) and *Morinda citrifolia* (Noni) on broiler gut health, showing reductions in pathogenic bacterial load and improvements in microbial balance. *C. aurantifolia* was also evaluated in aquatic species, with findings indicating comparable HT-related health benefits.

### Microbial inhibition and gut microbiota modulation

Across the 21 studies, tannin-rich extracts from Thai plants demonstrated inhibitory activity against 19 pathogenic bacterial species while promoting the proliferation of beneficial microbes (Table 4 [20–32, 34–40], supplementary data 6). The most commonly targeted pathogens were *E. coli* (12 studies) and *S. aureus* (10 studies). Extracts from *T. leontopetaloides*, *Q. infectoria*, and *F. indica* were particularly effective against these bacteria.

**Table 4:** Microbial inhibition and modulation by plant-based tannins, detailing pathogenic and beneficial bacteria, yeasts, and protozoa, along with the number of studies and plant names.

Microbes (number of species)	Microbial species	Number of Studies	Plant names
Pathogenic bacteria (n = 19)	*Escherichia coli*	12	Polynesian arrowroot [20], Vietnamese coriander [31], Wild Turmeric [34], Siamese cassia [38], Governor’s Plum [21], Pomegranate [30], Noni [35], Zingiber kerrii [25], Bitter bean [40], Mung bean [29], Cassava [32], Java plum and Mint [28]
	*Staphylococcus aureus*	10	Polynesian Arrowroot [20], Aleppo Oak [23], Bushwillows [27], Vietnamese coriander [31], Wild Turmeric [34], Governor’s Plum [21], Noni [35], Zingiber kerrii [25], Siamese Cassia [38], Bitter Bean [40]
	*Pseudomonas aeruginosa*	7	Wild turmeric [34], Siamese cassia [38], Governor’s plum [21], Noni [35], Zingiber kerrii [25], Mung bean [29], Shorea [22]
	*Bacillus subtilis*	4	Polynesian Arrowroot [20],Governor’s Plum [21], Zingiber kerrii [25], Siamese Cassia [38]
	*Bacillus cereus*	3	Wild Turmeric [34], Noni [35], Zingiber kerrii [25]
	*Vibrio parahaemolyticus*	3	Citrus [26], Pink water lily [36]
	*Listeria monocytogenes*	2	Wild turmeric [34], Mung bean [29]
	*Salmonella typhimurium*	2	Wild turmeric [34], Governor’s plum [21]
	*Staphylococcus epidermidis*	2	Noni [35], Zingiber kerrii [25]
	*Aeromonas hydrophila*	1	Pink water lily [36]
	*Vibrio vulnificus*	1	
	*Vibrio harveyi*	1	
	*Shigella sp.*	1	Siamese cassia [38]
	*Salmonella typhi*	1	
	*Klebsiella pneumoniae*	1	
	*Proteus mirabilis*	1	
	*Streptococcus pneumoniae*	1	
	*Proteus vulgaris*	1	Governor’s plum [21]
	*Serratia marcescens*	1	Zingiber kerrii [25]
Beneficial bacteria (n = 6)	*Lactobacillus plantarum*	2	Holy basil [39], Triphala [39]
	*Lactobacillus casei*	2	Holy basil [39], Triphala [39]
	*Lactobacillus rhamnosus*	1	Holy basil [39]
	*Bacteroides*	1	Triphala [39]
	*Bifidobacterium*	1	Mung bean [29], Triphala [39]
	*Faecalibacterium prausnitzii*	1	Mung bean [29]
Yeasts (n = 1)	*Candida albicans*	1	Governor’s plum [21]
Protozoa (n = 2)	*Eimeria spp.*	1	Kameng or false daisy [37]
	*Acanthamoeba triangularis*	1	Common tree-vine [24]

Other pathogens inhibited included *P. aeruginosa* (7 studies), *Bacillus subtilis* (4), *Bacillus cereus* (3), *Salmonella typhimurium* (2), and *Listeria monocytogenes* (2). In addition to bacteria, *Eclipta prostrata* (False daisy) inhibited *Eimeria* spp. Sporulation, relevant for coccidiosis control, and *F. indica* showed antifungal activity against *Candida albicans*.

In contrast to their inhibitory effects on pathogens, several extracts have been shown to enhance beneficial gut microbiota. *O. sanctum* (Holy basil) and Triphala formulations promoted *Lactobacillus plantarum* and *Lactobacillus casei*, whereas *Vigna radiata* (Mung bean) and *Phyllanthus emblica* (Indian gooseberry) increased the abundance of *Faecalibacterium prausnitzii*, a marker of gut health and anti-inflammatory potential.

### Economic feasibility and yield assessment

**Figure 2:**
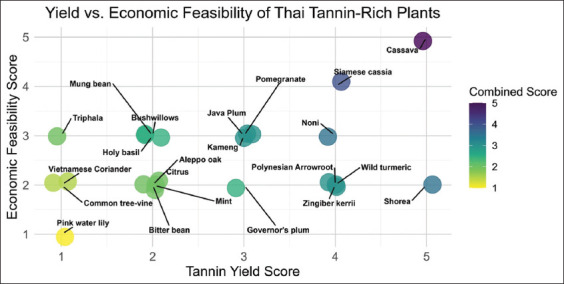
Economic feasibility and yield production assessment of Thai plants, with score size and color indicating the overall score from the assessment.

To determine the practical potential of these HT-rich plants in poultry feed, each species was evaluated using a scoring system based on two criteria: agricultural yield and economic feasibility, each rated on a scale from 1 (very low) to 5 (very high), as illustrated in Figure 2.

*M. esculenta* (Cassava) and *S. siamea* (*Siamese cassia*) received the highest combined scores, indicating excellent suitability for feed applications. Cassava leaves, a widely available agricultural by-product, stood out for their accessibility and cost-effectiveness. Although *S. siamea* scored slightly lower due to the labor-intensive harvesting of its leaves, its high tannin content and broad bioactivity profile supported its inclusion as a top candidate.

Plants with moderate feasibility scores included *M. citrifolia* (Noni), *S. roxburghii* (Shorea), *C. aromatica* (Wild turmeric), and *C. quadrangulare* (Bushwillows). These species offered acceptable yields but were limited by challenges such as primary use for non-feed purposes, niche demand, and difficult harvest logistics.

Conversely, species such as *Q. infectoria* (Aleppo oak), *Persicaria odorata* (Vietnamese coriander), and *Nymphaea pubescens* (Pink water lily) were deemed economically unfeasible for large-scale feed production due to low yields and poor commercial viability. Full scoring details are provided in Supplementary Data 5.

## DISCUSSION

This systematic review reveals the promising potential of HTs derived from Thai plants as natural feed additives in antibiotic-free poultry production. Numerous Thai plant species were found to exhibit potent antimicrobial, anti-inflammatory, antioxidant, and gut-modulating properties, positioning them as viable alternatives to conventional growth promoters.

### Antimicrobial properties and mechanisms of action

Gallic acid and ellagic acid were identified as the primary bioactive compounds responsible for the antimicrobial effects of HT-rich extracts. These tannins demonstrated inhibitory activity against 19 pathogenic bacterial species, with particular efficacy against *E. coli* and *S. aureus*. Plant species such as *T. leontopetaloides* (Polynesian arrowroot), *Q. infectoria* (Aleppo oak), and *F. indica* (Governor’s plum) exhibited strong antibacterial activity. In addition, *E. prostrata* (False daisy) inhibited *Eimeria* spp. Sporulation, showing antiparasitic potential, whereas *F. indica* also demonstrated antifungal activity against *C. albicans*.

Several extracts also influenced the gut microbiome. *M. esculenta* (Cassava) and *M. citrifolia* (Noni) improved broiler gut health by reducing pathogenic bacteria and enhancing beneficial microbial populations. Similar effects were reported in *P. emblica* (Indian gooseberry), *Terminalia bellirica* (Beleric myrobalan), and *Terminalia chebula* (Chebulic myrobalan)—key components of Triphala.

The antimicrobial action of tannins involves multiple mechanisms: iron chelation, disruption of bacterial membranes, inhibition of cell wall synthesis, and interference with fatty acid biosynthesis. Tannins also inhibit quorum sensing, disrupting virulence factors such as biofilm formation, enzyme secretion, motility, and toxin production [41, 42]. Recent advancements—such as tannin-loaded nanoparticles and hydrogels—further enhance their antibacterial potency and antivirulence efficacy [43]. While these findings are encouraging, further research is needed to assess their scalability in commercial poultry systems.

This evidence supports the use of plant-based tannins as natural alternatives to synthetic feed additives, particularly AGPs, which face growing restrictions due to AMR concerns. Previous studies by Redondo *et al*. [44] and Buyse *et al*. [45] on chestnut and quebracho tannins have shown significant improvements in poultry gut health and performance. Other sources, including Chinese gallnut tannic acid, have been shown to enhance gut microbiota in piglets [46], while *Acacia* and *Senna obtusifolia* have also demonstrated potential in poultry diets [47, 48]. Moreover, hydrolyzed gallotannins have been shown to improve immune and antioxidant functions in broilers [49]. Collectively, these findings highlight the role of gallic and ellagic acid as key agents in disease prevention in poultry, with *E. prostrata* showing particular promise in coccidiosis management [50, 51]. Additionally, *S. siamea* (*Siamese cassia*) and *P. granatum* (Pomegranate) provide antioxi-dant protection, enhancing resilience and perform-ance in poultry exposed to oxidative stress [52–54].

### Prebiotic effects and gut microbiome modulation

Beyond antimicrobial activity, tannins also exhibit significant prebiotic potential. Extracts from *Cassava* and *Noni* improved gut health in broilers by promoting microbial balance, which led to better feed conversion and growth performance. Similarly, *P. emblica*, *T. bellirica*, and *T. chebula* have been shown to positively influence gut microbiota by enhancing the populations of beneficial bacteria while suppressing those of harmful ones [55].

Tannins have been found to stimulate the proliferation of probiotic species, such as *L. plantarum*, *L. casei*, and *Bifidobacterium* spp., highlighting their potential role in improving digestion, immune function, and overall productivity [52, 56].

### Economic feasibility and local resource utilization

**Figure 3:**
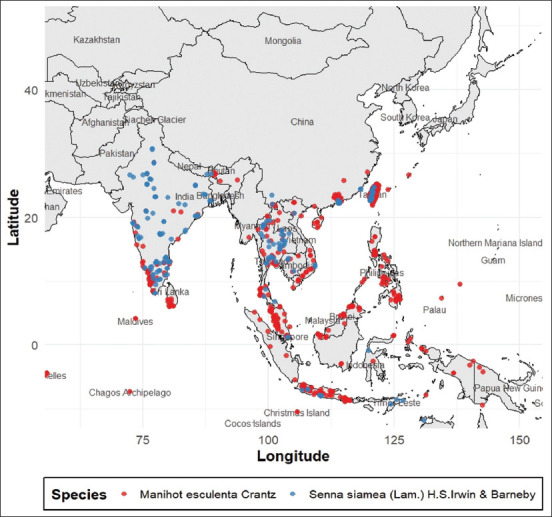
Geographical distribution of top candidate plants.

Economic feasibility is a critical factor in selecting alternative feed additives. The reliance on imported tannins from temperate species (e.g., chestnut and quebracho) substantially increases feed production costs. This review identifies *M. esculenta* (Cassava) and *S. siamea* (*Siamese cassia*) as the most viable local alternatives based on their high yield, affordability, and availability across Thailand and neighboring countries, as shown in Figure 3.

Cassava leaves, in particular, offer a low-cost and abundant resource, as they are typically treated as agricultural waste. Although concerns exist regarding cyanogenic compounds, existing research suggests that small quantities do not impair digestive enzyme activity in poultry [57]. Cassava leaves have already been studied as supplements in pig [58] and laying hen diets [59], further supporting their integration into poultry feed systems.

*S. siamea*, although slightly less feasible due to manual harvesting constraints, is widely cultivated across South and Southeast Asia and is traditionally used in animal feed. Its antioxidant and antimicrobial properties make it a promising candidate for local feed formulations [60].

### Limitations

Despite promising findings, several limitations must be acknowledged. First, there is a limited number of *in vivo* studies assessing the effects of HTs in poultry, with only two studies evaluating *C. aurantifolia* in aquatic models and *Cassava* in broilers. More controlled animal trials are needed to establish efficacy under real-world conditions.

In addition, most studies reported total phenolic content as gallic acid equivalents (mg GAE/g), which does not specifically represent HT content. Tannic acid equivalents (mg TAE/g) provide a more precise measure but are infrequently reported. Variability in extraction methods, including plant parts used, solvent types, and preparation formats, also complicates comparisons across studies and impacts reproducibility.

The variability in tannin composition, influenced by environmental factors, plant maturity, and processing techniques, further challenges consistent application. Standardization of extraction protocols and determination of optimal dosage ranges are urgently needed. Moreover, research on synergistic effects between tannins and other additives (e.g., probiotics, organic acids) may reveal enhanced feed efficacy and broaden the utility of these natural compounds.

### Future directions

To advance the application of HTs in poultry nutrition, future studies should:

- Conduct standardized phytochemical and antimicrobial profiling to quantify tannin content and bioactivity across species [61]
- Use whole-genome approaches to understand how tannins modulate bacterial gene expression, especially in gut pathogens [62]
- Implement field trials to assess long-term effects on broiler health, performance, and AMR under commercial settings [61]
- Develop cost-effective, scalable extraction techniques that preserve tannin functionality for feed-grade applications [63].

This review establishes the potential of Thai plant-derived HTs as sustainable and effective feed additives in poultry nutrition. While *in vivo* data remain limited, current evidence provides a strong foundation for the development of locally sourced, cost-efficient alternatives to synthetic growth promoters. By optimizing extraction protocols and feed incorporation strategies, these tannins can enhance poultry health and productivity while supporting antibiotic-free, environmentally sustainable agriculture.

## CONCLUSION

This systematic review highlights the potential of HTs derived from Thai plants as promising alternatives to conventional AGPs in poultry production. A total of 21 studies covering 24 native plant species demonstrated that HT-rich extracts exhibit broad-spectrum antimicrobial, antioxidant, anti-inflammatory, and gut-modulating effects. Notably, gallic acid and ellagic acid were the most frequently identified bioactive compounds, with strong inhibitory effects reported against *E. coli*, *S. aureus*, and *P. aeruginosa*. Extracts from *M. esculenta* (cassava) and *S. siamea* (*Siamese cassia*) emerged as top candidates due to their biological efficacy, high availability, and favorable cost-efficiency.

Practically, these findings support the use of locally sourced tannin-rich plant materials to reduce dependence on imported additives such as chestnut and quebracho tannins. The integration of cassava leaves, an agricultural by-product, into feed formulations offers a sustainable and low-cost strategy for enhancing poultry health and performance, particularly for smallholder farmers in tropical regions. In addition, several extracts showed potential for prebiotic modulation, promoting beneficial bacteria such as *Lactobacillus* spp. and *Bifidobacterium*, which are critical for nutrient assimilation and immune function.

The strength of this review lies in its systematic approach, adherence to PRISMA 2020 guidelines, and rigorous assessment of both biological efficacy and economic feasibility. The use of a validated scoring system to evaluate yield and cost potential adds practical value for policymakers and industry stakeholders.

However, the current evidence base is limited by the scarcity of *in vivo* trials and the lack of standardized quantification for HTs. Most studies reported total phenolic content (mg GAE/g), which does not accurately reflect HT-specific activity. Moreover, variability in extraction protocols hampers cross-study comparisons.

In conclusion, Thai plant-derived HTs, particularly from cassava and Siamese cassia, represent a viable path toward antibiotic-free poultry production. To fully realize their potential, future research should prioritize standardized extraction methods, controlled *in vivo* studies, and field-level performance trials. Such efforts will be essential for translating laboratory evidence into scalable, field-ready feed technologies that support animal health, reduce AMR, and advance sustainable livestock practices.

## DATA AVAILABILITY

Supplementary data can be made available from the corresponding author upon a request.

## AUTHORS’ CONTRIBUTIONS

TM: Conceptualization, investigation, methodology, review-first reviewer, and drafted and revised the manuscript. PP: Review–second reviewer. SNR: Data curation, data collection. TW: Conceptualization, supervision, and writing, review, and editing. TT: Conceptualization, review–third reviewer, and writing–review and editing. All authors have read and approved the final manuscript.
